# Fibromyalgia and Inflammation: Unrevealing the Connection

**DOI:** 10.3390/cells14040271

**Published:** 2025-02-13

**Authors:** Mario García-Domínguez

**Affiliations:** 1 Program of Immunology and Immunotherapy, CIMA-Universidad de Navarra, 31008 Pamplona, Spain; mgdom@unav.es; 2 Department of Immunology and Immunotherapy, Clínica Universidad de Navarra, 31008 Pamplona, Spain; 3 Centro de Investigación Biomédica en Red de Cáncer (CIBERONC), 28029 Madrid, Spain

**Keywords:** fibromyalgia, peripheral and central inflammation, pain, neurogenic inflammation, dietary habits, inflammatory markers

## Abstract

Fibromyalgia represents a chronic pain pathology characterized by severe musculoskeletal pain, fatigue, disturbances in sleep, and cognitive issues. Despite its presence, the underlying mechanisms of fibromyalgia remain inadequately understood; however, recent investigations have suggested that inflammation could play a fundamental role in the pathophysiology of this condition. Several studies highlight elevated concentrations of pro-inflammatory cytokines, dysregulation of immune responses, and neuroinflammation in fibromyalgia patients. Furthermore, chronic low-grade inflammation has been proposed as a potential catalyst for the sensitization of pain pathways, which exacerbates the symptoms of fibromyalgia. Understanding the role of inflammation in this disease might open new avenues for therapeutic interventions while providing a more profound insight into the complex nature of this debilitating disorder. Although progress has been made, further research is needed to uncover the complexities involved. This review investigates the intricate relationship between inflammation and fibromyalgia, analyzing the evidence that supports the involvement of both peripheral and central inflammatory processes in the onset and persistence of the disorder.

## 1. Introduction

Fibromyalgia represents a chronic condition characterized by widespread musculoskeletal pain, frequently accompanied by a plethora of symptoms that profoundly reduce the overall quality of life [1]. This disorder is estimated to impact between 0.2 and 6.6% of the global population, with a noticeable predominance among women [2,3]. The hallmark manifestations of fibromyalgia encompass persistent discomfort throughout the body, debilitating fatigue, sleep disturbances, and cognitive impairments (known as “fibro-fog”) [4]. Patients frequently report heightened sensitivity to pain (allodynia and hyperalgesia), stiffness in muscles and joints, headaches, and irritable bowel syndrome (IBS), along with mood disorders such as anxiety and depression [4]. Although the exact cause of fibromyalgia remains poorly understood, researchers postulate that it might stem from aberrant pain processing within the central nervous system (CNS), potentially prompted by several factors such as physical trauma, infections, stress, and genetic predispositions [5]. The diagnosis is especially challenging due to the lack of definitive tests and the significant overlap of symptoms with those of other conditions [6]. Although a definitive treatment for fibromyalgia has not been established, treatment often necessitates a multifaceted approach that includes lifestyle changes and pharmacological agents, such as nonsteroidal anti-inflammatory drugs (NSAIDs), to manage symptoms [7]. Despite its challenges, effective management strategies enable many individuals with fibromyalgia to maintain their fulfilling lives and continue their daily activities.

Inflammation in the CNS (known as central inflammation) has been identified as a contributing factor in the development of fibromyalgia [8]. Several studies have shown elevated levels of pro-inflammatory cytokines in the cerebrospinal fluid (CSF) of fibromyalgia patients [9,10]. Moreover, brain imaging techniques have revealed strong activation of the glial cells in large parts of the CNS in fibromyalgia patients [11,12]. The degree of glial cell activation has been highly correlated with the severity of fatigue reported by the patients, suggesting a potential link between neuroinflammation and symptom manifestation [13]. In addition to central inflammation, peripheral mechanisms (known as *peripheral inflammation*) also participate in fibromyalgia pathogenesis [14]. Evidence suggests that nociceptors placed in the skin and muscles experience changes, such as sensitization of vanilloid receptors (TRPVs), acid-sensing ion channel receptors (ASICs), and purinergic receptors [15,16]. These changes in peripheral pain processing contribute to pain sensitivity typical of the symptomatology associated with fibromyalgia.

The interplay between peripheral and central mechanisms is very complex, with peripheral stimulation potentially initiating and maintaining central sensitization. However, most researchers believe that the CNS plays essential roles in modulating peripheral sensory inputs, leading to amplification of pain signals in fibromyalgia [16]. The central sensitization process results in *neurogenic inflammation*, which contributes to several hallmark features of fibromyalgia, including abnormal tenderness, pain, peripheral swelling, and cognitive dysfunction [17,18].

This article examines the complex relationship between inflammation and fibromyalgia, investigating the potential role of inflammatory mechanisms in the onset and progression of this multifaceted condition. The article emphasizes substantial evidence from studies showing increased levels of pro-inflammatory markers in individuals with fibromyalgia, indicating a significant link between inflammation and the development of pain, fatigue, and other debilitating symptoms. By elucidating these associations, this review underscores the promise of pioneering therapeutic approaches designed to target inflammatory pathways. Such advancements could change the management of symptoms and substantially improve the overall quality of life for individuals with fibromyalgia.

## 2. Fibromyalgia: A Comprehensive Overview

In this section, the general characteristics of fibromyalgia will be thoroughly examined, focusing on its symptomatology, diagnostic criteria, and epidemiology. It examines the clinical manifestations of the condition, the approaches and challenges in diagnosing it, and the prevalence trends across various populations. Through the exploration of these aspects, this section aims to provide a clearer understanding of the complexity of fibromyalgia and its impact on both individuals and public health.

### 2.1. Signs and Symptoms

Fibromyalgia constitutes a complex disorder characterized by persistent, widespread musculoskeletal pain, particularly affecting lumbar, gluteal, cervical, scapular, and dorsal regions [2,4,19]. Pain can also extend to the head and occasionally to the limbs; however, peripheral pain is less common [2,4,19]. Diagnosing fibromyalgia requires consideration of the interplay of symptoms, as any misinterpretation could lead to improper treatments [20]. Pain exhibits variability over time, influenced by factors including seasonal changes and physical activity levels; in fact, inactivity has been found to intensify symptoms [20]. Moreover, fibromyalgia usually evokes severe fatigue, sleep disturbances, and cognitive dysfunctions, which can significantly disrupt daily functioning, highlighting the need for treatments that target both musculoskeletal and systemic pain-related symptoms [21,22,23]. Finally, stress, humidity, and low temperatures increase the sensation of pain [7].

### 2.2. Diagnosis

Prior to 2010, the diagnosis was established based on the American College of Rheumatology (ACR) 1990 criteria, which required documentation of widespread pain lasting at least three months and the detection of *tender points* [24]. Although tender points remain a vital component of a comprehensive clinical evaluation, these criteria were comprehensively revised in 2016 to reduce the likelihood of misdiagnosing fibromyalgia [25]. However, differences in individual phenotypes and the presence of concurrent pathologies can result in incomplete clinical evaluations, which might compromise diagnostic accuracy. In this respect, the probability of defining an accepted diagnostic criterion is problematic [26].

In 2020, the ACR revised the 2016 criteria to highlight two key parameters focusing on somatic and cognitive symptoms [27]: the Widespread Pain Index (WPI) and the Symptom Severity (SS) score. The WPI evaluates pain across 19 anatomical regions [28], while the SS score assesses severity of symptoms such as fatigue, sleep disturbances, and cognitive impairments [29]. Although these revisions improve diagnostic accuracy, they require a comprehensive understanding of individual presentations [27].

Currently, no specific biomarkers have been identified for diagnosing fibromyalgia. As a result, most ongoing research studies are focused on the search for diagnostic markers by exploring genetic, environmental, and epigenetic variables that influence the pathophysiology of fibromyalgia [30].

### 2.3. Epidemiology and Socioeconomic Impact

Understanding the epidemiology of fibromyalgia offers clinical and financial benefits [31]. Prevalence rates exhibit significant variability, originating from differences in methodology, diagnostic criteria, and geographic location [32]. Most studies are primarily focused on specific cities and regions, resulting in a lack of comprehensive data on nationwide prevalence [33].

Global estimates range from 0.2% to 6.6% [3], with Europe reporting a prevalence of 2.31% [34]. In specific countries, prevalence rates are 1.6% in France [35], 2.1% in Germany [36], and 2.4% in Spain [37]. Other countries, such as the United States, Canada, and Japan, report prevalence rates of 6.4%, 1.5%, and 2.1%, respectively [38,39,40]. This disease is more prevalent among females and tends to escalate with age [41,42]. Additionally, additional factors associated with an increased likelihood of developing fibromyalgia include diagnoses of major depressive disorder, IBS, and restless legs syndrome (RLS) [43,44].

On the other hand, fibromyalgia significantly affects society in both clinical and economic costs. Individuals with lower socioeconomic status frequently encounter more pronounced symptoms, reduced productivity, and elevated absenteeism, all contributing to the economic burden of ill patients [45,46,47]. The healthcare costs associated with this condition are considerable; patients usually necessitate many treatments and consultations to manage their symptoms [48]. The nature of this condition leads to accumulating costs over time, creating a substantial financial strain on patients, their corresponding families, and healthcare systems [48]. Addressing these problems remains challenging because the interplay between health and socioeconomic factors is complex. Although interventions can mitigate numerous effects, the overarching impact of fibromyalgia on economic stability cannot be overlooked.

The nature of fibromyalgia results in delays in both diagnosis and treatments, consequently intensifying its socioeconomic repercussions [49]. The psychological impact of fibromyalgia can substantially decrease the quality of life and social participation, which, in turn, may indirectly influence economic outcomes [50]. Consequently, the diverse socioeconomic consequences of fibromyalgia outline the importance of complex treatment approaches to cover medical and social aspects of the disease [7]. Still, further research has an urgent need to enhance the knowledge of such interrelations.

### 2.4. Current Pharmacological Treatments

A significant body of research has clarified a variety of pharmacological approaches used as supportive therapies in the management of fibromyalgia. These interventions include antidepressants, monoamine reuptake inhibitors, serotonin and dopamine receptor antagonists, gabapentinoids, opioids, and cannabinoids. The main goal of these pharmacological agents is to improve sleep quality, alleviate symptoms of depression and anxiety, and decrease fatigue. While each intervention functions through distinct mechanisms of action, their aim is to improve the overall quality of life for patients. The pharmacological agents employed in the management of fibromyalgia are outlined as follows (Table 1):

## 3. The Relationship Between Fibromyalgia and Inflammation

The relationship between fibromyalgia and inflammation represents a multifaceted and dynamic area of investigation, highlighting the sophisticated interplay between the immune system and the nervous system [120]. While fibromyalgia has traditionally been classified as a disorder primarily characterized by CNS sensitization, recent evidence increasingly emphasizes the role of inflammation in its pathophysiology [121,122]. The inflammatory milieu is thought to exacerbate the increased sensitivity to pain that typifies this condition, because it promotes peripheral nociceptor sensitization while amplifying central sensitization processes [121]. Furthermore, neuroinflammation (the interplay between immune cells and the nervous system) may worsen symptoms, creating a feedback loop that perpetuates pain [122].

Although the complexity of this relationship is evident, it underscores the need for a multidisciplinary approach to fully understand this connection. Given these findings, researchers and clinicians must work together to uncover the underlying mechanisms.

### 3.1. Peripheral Inflammation and Fibromyalgia

The recent evidence underscores the pivotal role of peripheral inflammation in the pathophysiology of fibromyalgia, indicating that inflammation is not simply a result of fibromyalgia but a fundamental factor driving its development and ongoing persistence [121]. Some studies, employing the ELISA method, have demonstrated that fibromyalgia patients often exhibit elevated levels of inflammatory markers (Table 2), including C-reactive protein (CRP), which serves as a good indicator of low-grade systemic inflammation [123,124,125]. However, not all individuals with fibromyalgia manifest elevated CRP levels; in fact, the extent of elevation can differ markedly across patients. Some studies have indicated that CRP concentrations in individuals with fibromyalgia may be influenced by comorbid conditions, such as obesity or concurrent inflammatory disorders, highly prevalent within this cohort [126,127]. This inflammatory state can begin a catalytic effect, amplifying the painful symptoms that characterize fibromyalgia and potentially exacerbating the overall burden of the condition [128]. Such complexity requires further investigation, as understanding these relationships is vital for developing effective treatment strategies. CCL2, a chemokine involved in the inflammation process [129], has been identified in the plasma of patients with fibromyalgia using the ELISA method [9].

On the other hand, some studies have identified the interferon gene signature in fibromyalgia patients. This signature is characterized by the increased expression of interferon-regulated genes in peripheral B cells, as assessed by RNA sequencing and RT-qPCR. These genes, including *S100A8*, *S100A9*, *VCAM*, *CD163*, *SERPINA1*, and *ANXA1*, are involved in immune response and inflammation [14]. The elevated expression of these genes demonstrates that fibromyalgia might involve an autoimmune-like aspect, with the immune system targeting the body’s own tissues [139].

A recent publication showed the proteomes of plasma, serum, and saliva in healthy individuals and fibromyalgia patients. The most significant proteins identified in patients with fibromyalgia included transferrin; α-, β-, and γ-fibrinogen chains; profilin-1; transaldolase; PGAM1; apolipoprotein-C3; complement C4A and C1QC; immunoglobulin components; and acute phase reactants [130]. Some of these are implicated in the maintenance of chronic low-grade inflammation [140,141,142,143]. Moreover, increased levels of inflammatory serum proteins, including IL-8, IL-37, AXIN1, and SIRT2, have been identified through proteomics and ELISA as correlates of fibromyalgia symptom severity [14,144]. IL-8 and IL-37 participate in the recruitment of some immune cells to inflammation sites. Consequently, its elevated levels show a highly active inflammatory response in patients with fibromyalgia [145,146]. Similarly, AXIN1 and SIRT2 are linked to immune regulation and cellular stress and have been linked to the severity of pain, fatigue, and some other symptoms in fibromyalgia [147,148]. Other investigations that analyzed plasma proteins using ELISA have reported elevated levels (pro-inflammatory cytokines) alongside reductions in IL-4 and IL-13 (anti-inflammatory cytokines), thereby inducing the activation of various immune cells, including mast cells [131,132,133,134,135]. In this regard, fibromyalgia patients have displayed, as assessed by flow cytometry, an increased neutrophil/lymphocyte ratio and alterations in several T lymphocyte subpopulations, including CD4^+^ T cells and NKT cells [149,150]. All the aforementioned biomarkers provide important insights into the inflammatory processes involved in fibromyalgia, thereby serving as potential targets for the development of future diagnostic tools [151,152].

**Figure 1:**
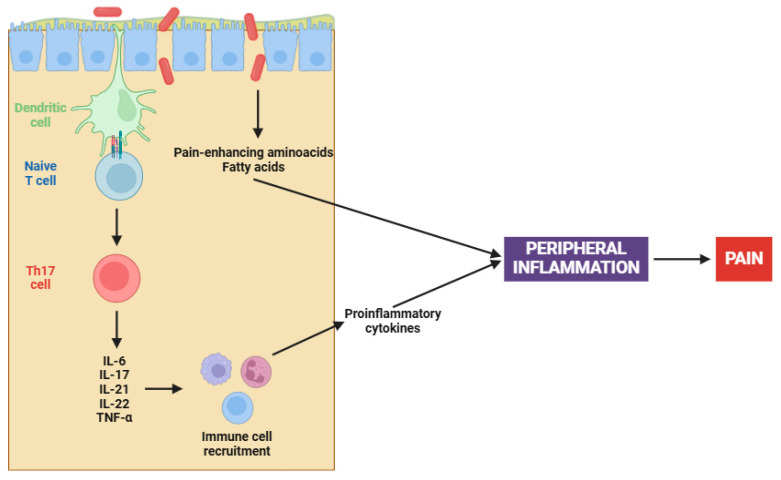
Gut dysbiosis in fibromyalgia patients. Abbreviations: IL-6 (interleukin 6), IL-17 (interleukin 17), IL-21 (interleukin 21), IL-22 (interleukin 22), and TNF-α (tumor necrosis factor alpha).

On the other hand, the complex interaction between gut microbiota and fibromyalgia has emerged as a central focus in elucidating the pathophysiology of this chronic condition [153,154]. Recent studies have illuminated a compelling causal nexus between altered gut microbiota and fibromyalgia symptoms, particularly in relation to peripheral sensitization [155]. Pioneering research has demonstrated that fecal microbiota transplantation (FMT) from patients with fibromyalgia into germ-free mice induces pain hypersensitivity; however, transplantation from healthy individuals has been ineffective in reproducing this phenomenon [156,157]. Certainly, this involves an increase in pro-inflammatory cytokines (e.g., IL-17 and TNF-α) and the activation of monocytes and lymphocytes, both contributing to the peripheral sensitization process (Figure 1) [156,157].

The altered microbiota in fibromyalgia patients is characterized by imbalances in certain bacterial species, such as *Flavonifractor plautii*, *Parabacteroides merdae*, and *Faecalibacterium prausnitzii* [158]. Apart from cytokine participation, microbial imbalances contribute to peripheral sensitization through several mechanisms, including the synthesis of pain-enhancing amino acids (e.g., glutamate), alterations in fat metabolism, and changes in bile acid production [159]. Chronic immune activation, when coupled with an altered gut microbiota, results in increased intestinal permeability. This ultimately leads to further activation of the immune system and initiates an inflammatory cascade that stimulates nociceptive signals [156,157].

The connection between peripheral inflammation and fibromyalgia extends beyond pain perception; it also notably influences the comorbid conditions commonly seen in individuals with fibromyalgia [160,161]. Numerous conditions, including inflammatory arthritis, chronic spontaneous urticaria (CSU), and functional bowel disorders (FBD), are usually encountered alongside fibromyalgia [160,161]. These conditions suggest the presence of an underlying inflammatory pathway may be involved, impacting various organ systems and intensifying the overall symptom burden [162,163,164]. Continuous activation of the immune system in these conditions perpetuates the inflammatory state, therefore underscoring the complex nature of fibromyalgia [160,161].

In conclusion, the interplay between peripheral inflammation and fibromyalgia underscores the importance of inflammation as a key factor in the onset and progression of the disease. Inflammatory cytokines and activation of the immune cells in the periphery can directly affect the function of the nervous system, enhancing excitability of pain pathways and leading to an exaggerated pain response. Consequently, a self-sustaining feedback loop is established, where pain, inflammation, and immune dysfunction reinforce each other. However, these interconnections complicate the understanding of the underlying mechanisms.

### 3.2. Central Inflammation and Fibromyalgia

**Figure 2:**
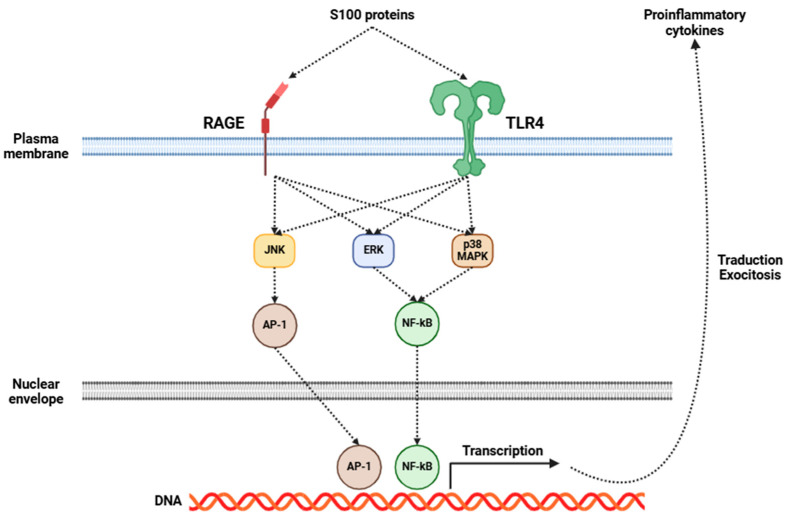
Mechanisms of action triggered by the activation of RAGE and TLR4 receptors in glial cells by S100 proteins. This activation leads to the release of pro-inflammatory cytokines from these cells. Abbreviations: RAGE (receptor for advanced glycation endproducts), TLR4 (Toll-like receptor 4), JNK (c-Jun N-terminal kinase), ERK (extracellular-signal-regulated kinase), p38 MAPK (p38 mitogen-activated protein kinase), AP-1 (activator protein 1), NF-κB (nuclear factor kappa-light-chain-enhancer of activated B cells), and DNA (deoxyribonucleic acid).

Fibromyalgia has been increasingly recognized as a disorder intricately connected to central inflammation, with neuroinflammation emerging as an essential component of its pathophysiology [5,165,166]. Central to this process is the activation of glial cells, particularly microglia and astrocytes. Upon activation, these cells initiate a series of inflammatory events, resulting in the release of many pro-inflammatory cytokines, including IL-1β, IL-6, IL-8, IL-10, TNF-α, BDNF, and GDNF, among others [167,168]. Several investigations have shown elevated levels of some of the aforementioned cytokines in the CSF of fibromyalgia patients (Table 2), indicating a pervasive state of central inflammation [136,137,138,169]. Moreover, recent studies have emphasized the significance of S100 proteins in the field of fibromyalgia research [170]. These proteins are involved in many inflammatory processes, and they may exert a considerable influence on fibromyalgia’s development and progression. The role of S100 proteins in fibromyalgia may be mediated through RAGE and TLR4, which, in turn, activate signaling pathways that promote the release of several pro-inflammatory cytokines (Figure 2), as previously mentioned [170].

Central sensitization enhances pain signaling, converting otherwise innocuous stimuli into significant sources of discomfort and contributing to the chronic and widespread pain characteristic of fibromyalgia. In addition to pain amplification, dysregulation of inflammatory processes leads to an imbalance between pro-inflammatory and anti-inflammatory cytokines [171]. As a result, the neuroinflammatory state is sustained, establishing a feedback loop that further worsens the condition [8]. Neuroimaging studies have further corroborated these findings, revealing microglial activation in patients with fibromyalgia [8,11]. The consequences of this persistent neuroinflammatory state extend beyond pain hypersensitivity, playing a key role in the development of a broad spectrum of debilitating symptoms commonly associated with fibromyalgia [172]. Cognitive dysfunction, often referred to as “fibro fog”, serves as an additional manifestation of this inflammatory state, likely stemming from the detrimental effects of cytokines on neural connectivity and synaptic function [173]. Sleep disturbances, which are both a symptom and a contributing factor to fibromyalgia, may be intricately linked to the inflammatory process, as cytokines have the ability to affect sleep regulation and disrupt restorative sleep cycles [174,175].

**Figure 3:**
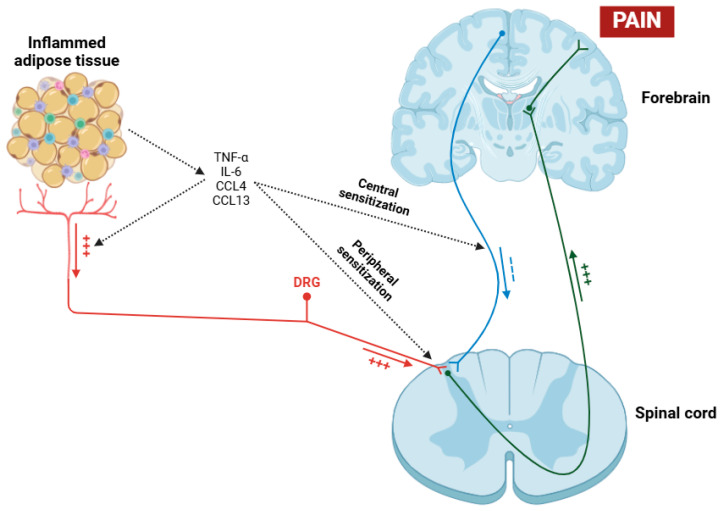
Pathological process in which obesity acts as a contributing factor to hyperalgesia in patients with fibromyalgia. Through the mechanisms of peripheral and central sensitization induced by the action of various cytokines (TNF-α, IL-6, CCL4, and CCL13), nociceptive signal transmission to the cerebral cortex is facilitated, while descending inhibitory signals are suppressed. Abbreviations: TNF-α (tumor necrosis factor alpha), IL-6 (interleukin 6), CCL4 (chemokine (C-C motif) ligand 4), CCL13 (chemokine (C-C motif) ligand 13), and DRG (dorsal root ganglia).

On the other hand, obesity exerts a profound and multifaceted influence on central inflammation in fibromyalgia patients [176,177]. The relationship between obesity and fibromyalgia is complex and bidirectional, with obesity potentially serving as a risk factor and an aggravating factor for this condition [178]. Obesity is involved in central inflammation through some mechanisms, primarily the secretion of pro-inflammatory cytokines and chemokines such as TNF-α, IL-6, CCL4, and CCL13 by adipose tissue, primarily due to macrophages [179,180,181,182]. These cytokines, in addition to exerting their effects on the sensitization of nociceptors and dorsal root ganglia (DRGs) [183], can cross the blood–brain barrier (BBB) and have the potential to activate microglial cells placed in the CNS, thereby maintaining a state of neuroinflammation (Figure 3) [184,185]. Moreover, a recent study has shown that obesity in fibromyalgia patients acts as a disruptor of the descending pain pathway, thereby exacerbating symptoms associated with this condition [186].

Obesity is closely linked to metabolic dysregulation, including insulin resistance and leptin resistance, both of which play a role in the modulation of pain perception and neuroinflammation [187]. Higher levels of leptin have been found to correlate with increased pain sensitivity and inflammatory markers in fibromyalgia patients, suggesting a direct relationship between adiposity and central pain processing [188,189]. Furthermore, obesity exacerbates systemic low-grade inflammation, which amplifies central sensitization processes characteristic of fibromyalgia, establishing a vicious cycle in which pain and inflammation sustain each other [190]. The comorbidity of obesity and fibromyalgia is also linked to worsened clinical outcomes (such as higher pain intensity) and reduced physical function; however, increased fatigue can further compromise quality of life [191]. In addition to these effects, obesity usually results in sleep disturbances and obstructive sleep apnea, both of which are prevalent in fibromyalgia and contribute to the further disruption of inflammatory and neuroendocrine pathways [176].

Common behavioral and lifestyle factors, including physical inactivity and poor dietary habits, also contribute to both conditions, perpetuating the inflammatory state and hindering effective management [190]. Given this interplay, addressing obesity in fibromyalgia patients through weight management, anti-inflammatory interventions, and lifestyle modifications may offer a promising therapeutic approach to mitigate central inflammation, reduce symptom severity, and improve overall health outcomes. Nevertheless, it is very important to recognize the complexity of these interactions, as they have a significant impact on treatment effectiveness. Although these factors are crucial, additional underlying mechanisms must also be explored to obtain a thorough understanding of their impact on patient health.

## 4. Current Anti-Inflammatory Strategies for Fibromyalgia

Contemporary anti-inflammatory interventions for fibromyalgia condense a diverse array of pharmacological and non-pharmacological therapies. Clinical guidelines recommend low-dose acetaminophen and NSAIDs for managing some chronic pain conditions linked to fibromyalgia [192,193,194]. Although typically used, no Cochrane review supports the effectiveness of acetaminophen for fibromyalgia. In contrast, a Cochrane review of six randomized controlled trials (RCTs) found that NSAIDs did not provide a significant pain-relief benefit compared to placebo [195]. Moreover, the European League Against Rheumatism (EULAR) does not recommend the use of NSAIDs [196].

Fibromyalgia is classified as a condition linked to low-grade inflammation. This level of inflammation is likely too mild for NSAID treatments to be effective on their own [197]. However, NSAIDs are more effective in patients with fibromyalgia who also have comorbid inflammatory conditions, like osteoarthritis [192], migraine [193], and rheumatoid arthritis [194]. This is due to the presence of a higher degree of inflammation, which is substantial enough for NSAIDs to be highly effective. In such cases, the NSAIDs can target the more pronounced inflammatory processes, leading to greater symptom relief [198]. As a result, this enhanced efficacy not only helps reduce the pain associated with the comorbid condition but also contributes to an overall improvement in the symptoms of fibromyalgia.

Several NSAIDs, including ibuprofen, naproxen, and aspirin, are usually advocated for pain management, although their prolonged administration poses inherent risks, such as gastrointestinal hemorrhaging and cardiovascular problems [199]. Administration of NSAIDs, for example, celecoxib, is within the context of combination therapies that synergize with the antiviral agent famciclovir. This strategy has demonstrated potential in some clinical studies aimed at reducing fibromyalgia-related pain and fatigue [200].

Non-pharmacological therapies, including dietary modifications, play a crucial role in managing fibromyalgia and are typically regarded as first-line treatments due to their positive safety profiles and potential for long-term benefits [201]. Among these therapies, dietary modifications are gaining recognition as vital components of an anti-inflammatory strategy for managing fibromyalgia [202,203]. Dietary changes (e.g., anti-inflammatory diets) have yielded prominent results in the management of fibromyalgia symptoms [204,205]. Novel investigations have examined many dietary strategies, including an anti-inflammatory FODMAP diet, which resulted in better patient-reported outcomes, including reduced pain, fatigue, and gastrointestinal challenges. This diet excludes gluten, dairy, sugar, and ultra-processed foods [206]. A personalized Mediterranean diet has also shown affirmative effects on pain reduction and quality of life enhancement in fibromyalgia patients [207]. Furthermore, a gluten-free (FODMAP) and low histamine diet (IGUBAC-Diet^®^), when combined with an olive-tree-based supplement, exhibited advantageous effects on the severity of fibromyalgia symptoms [208]. On the other hand, while many research have studied the potential benefits of supplementation with antioxidants, magnesium, CoQ_10_, and vitamins C and D, the management of symptomatology remains a complex domain [209,210,211,212,213,214]. In fact, the Dietary Inflammatory Index (DII) has been correlated with pressure pain thresholds in fibromyalgia patients; suggesting that a diet with a reduced inflammatory profile may help mitigate pain hypersensitivity [204].

These dietary interventions, aimed at reducing inflammation, are designed to modulate the gut microbiome and address potential nutrient deficiencies that may contribute to fibromyalgia symptoms [215]. Although further research is required to establish definitive dietary guidelines for fibromyalgia management, the available evidence suggests that an anti-inflammatory diet approach may constitute a valuable component of comprehensive treatment plans for individuals with fibromyalgia [216]. However, it is crucial to recognize that this approach is not universally applicable, because individual responses to dietary changes can vary significantly [217].

Other significant non-pharmacological therapies include physical exercise, particularly aerobic and strength training programs, which have been proven effective in reducing symptoms such as pain, sleep disturbances, fatigue, and depression [218]. Aerobic exercise enhances pain relief and functionality by stimulating endogenous opioid pathways and promoting the activation of opioid receptors in the CNS [219]. Regular exercise also promotes an increase in β-END synthesis, which exerts an anti-inflammatory effect and contributes to increase pain thresholds [220,221,222].

Although these interventions are supported by a growing body of evidence, the quality of studies varies significantly, underscoring the necessity for further rigorous research [223]. However, the existing evidence underscores the great importance of incorporating non-pharmacological treatments into a multidisciplinary approach to fibromyalgia care, as they can notably improve patient outcomes [224].

## 5. Conclusions

Fibromyalgia is a chronic condition marked by severe musculoskeletal pain, fatigue, sleep disturbances, and cognitive impairments. Numerous investigations suggest that inflammation plays a fundamental role in the onset and development of this condition. On the other hand, fibromyalgia is characterized by increased contents of pro-inflammatory cytokines in some biological fluids (e.g., plasma and serum), immune dysregulation, and neuroinflammation processes. Chronic low-grade inflammation appears to contribute significantly to the sensitization of pain pathways, thereby exacerbating fibromyalgia symptoms.

This review offers a comprehensive analysis of the role of inflammation in the pathophysiology of fibromyalgia, clarifying its significant contribution to chronic pain experienced by affected individuals. In addition to explaining several characteristics of this pathology, this paper highlights that both peripheral and central inflammation processes are crucial factors in the initiation and progression of fibromyalgia symptoms. Specifically, it emphasizes the influence of pro-inflammatory cytokines in the development of neuroinflammation and how this biological phenomenon contributes significantly to the dysregulation of pain processing pathways, amplifying the obstacles experienced by individuals with this disease. Although the complexity of the mechanisms involved seems formidable, the relationship between inflammation and pain is defined by a strong interaction: chronic pain exacerbates inflammatory responses, and these persistent inflammatory processes, in turn, intensify pain sensation. This vicious cycle significantly contributes to the debilitating symptoms of fibromyalgia; however, understanding it is crucial for developing effective therapeutic strategies. In summary, future research could investigate the function of inflammatory markers in patients with fibromyalgia, aiming to determine whether they contribute to the peripheral and central sensitization that defines this condition. Additionally, investigating the impact of many environmental factors, genetic predispositions, and comorbid conditions on the inflammatory response in fibromyalgia will be essential for advancing personalized treatment approaches.

On the other hand, the utilization of NSAIDs in fibromyalgia has been a subject of continuous research and clinical investigation. Although fibromyalgia is associated with an inflammatory component, studies indicate that NSAIDs alone are not significantly effective in managing the condition. This limited efficacy implies that the inflammation associated with fibromyalgia may differ in both complexity and nature from the type typically targeted by NSAIDs. However, the identification of a potential inflammatory component in fibromyalgia has driven the advancement of new therapeutic strategies. While NSAIDs might not offer a definitive solution, the focus on inflammation has motivated researchers to explore more targeted approaches to modulate the inflammatory response in fibromyalgia patients.

Finally, future research will focus on identifying specific subgroups of fibromyalgia patients who may derive greater benefits from NSAIDs while also exploring the potential integration of these drugs with other therapies. Additionally, investigations are expected to examine how NSAIDs may influence neuroinflammatory processes that are believed to play a fundamental role in the development of fibromyalgia symptoms. However, current research on the efficacy of NSAIDs for fibromyalgia is limited, particularly due to the lack of long-term studies. Longitudinal research is crucial to understanding the long-term effects of NSAIDs on fibromyalgia, aiding informed treatment decisions.

## Figures and Tables

**Table 1:** A list of drugs used in the management of pain-related symptoms of fibromyalgia. Abbreviations: TCAs (tricyclic antidepressants); 5-HT (serotonin); NA (noradrenaline); NMDA (N-Methyl-D-aspartate); SNRIs (serotonin-noradrenaline reuptake inhibitors); CNS (central nervous system); NRIs (selective noradrenaline reuptake inhibitors); SSRIs (selective serotonin reuptake inhibitors); 5-HT2A (serotonin 5-HT2A receptor); 5-HT2C (serotonin 5-HT2C receptor); 5-HT3 (serotonin 5-HT3 receptor); D_2_ (dopamine D_2_ receptor); VGCCs (voltage-gated calcium channels); MOR (μ-opioid receptor); CB1 (cannabinoid receptor 1); CB1 (cannabinoid receptor 2).

Drug Class	Drugs	Mechanisms of Action	Side Effects	References
TCAs	Amitryptiline Nortriptyline Doxepin	Modulation of 5-HT and NA neurotransmission Impact on potassium channels and NMDA receptors	Dry mouth, constipation, and drowsiness	[51,52,53,54,55]
SNRIs	Duloxetine Milnacipran	Raising the levels of 5-HT and NA in the CNS	Nausea and drowsiness	[56,57,58,59]
NRIs	Reboxetine Esreboxetine Atomoxetine	Boosting the levels of 5-HT and NA in the CNS	Headache, dry mouth, abdominal pain, nausea, and insomnia	[60,61,62,63]
SSRIs	Citalopram Escitalopram Fluoxetine Paroxetine Sertraline	Elevating the concentration of 5-HT in the CNS	Nausea, dyspepsia, anorexia, dizziness, blurring of vision, dry mouth, sweating, sleep disturbance, headache, and sexual dysfunction	[64,65,66,67,68,69,70,71]
5-HT receptor antagonists	Cyclobenzaprine Tropisetron Trazodone	Blockade of 5-HT2A, 5-HT2C, and 5-HT3 receptors	Nausea, dizziness, xerostomia, constipation, drowsiness, alterations in mood, blurred vision, and problems with concentration	[72,73,74,75,76]
Gabapentinoids	Pregabalin Gabapentin Mirogabalin	Blockade of the α2δ subunit of VGCCs	Dizziness, somnolence, peripheral edema, weight gain, cognitive impairment, and increased suicide risk	[77,78,79,80,81,82]
Antipsychotics	Quetiapine Olanzapine	Blockade of 5-HT2A and dopamine D_2_ receptors	Weight gain and somnolence	[83,84,85,86]
Dopamine receptor agonists	Pramipexole Ropinirole Rotigotine	Activation of dopamine receptors	Nausea, vomiting, orthostatic hypotension, sleep issues, weight loss, gastrointestinal problems, and impulse control disorders	[87,88,89,90,91]
Opioids	Tramadol Tapentadol Hydrocodone Codein Fentanyl Naltrexone	MOR agonist and SNRI agonist MOR agonist with NRI activity MOR agonist MOR agonist MOR agonist Non-selective opioid antagonist	Nausea, headaches, insomnia, dizziness, constipation, somnolence, respiratory depression, seizures, cardiovascular effects, risk of dependence, and withdrawal symptoms	[92,93,94,95,96,97,98,99,100,101,102,103,104,105]
NMDA receptor antagonists	Ketamine Memantine	Blockade of NMDA receptors	Hypertension, confusion, headache, constipation, cough, generalized pain, yawning, vomiting, and dyspnea	[106,107,108,109]
Cannabinoids	Nabilone Dronabinol Ajulemic acid	Activation of CB1 and CB2 receptors	Euphoria, tachycardia, hypotension, tolerance development, and paranoia	[110,111,112,113,114,115,116,117,118,119]

**Table 2:** List of biomarkers with significant differences in patients with fibromyalgia. Abbreviations: CRP (C-reactive protein), CCL2 (chemokine (C-C motif) ligand 2), S100A8 (S100 calcium-binding protein A8), S100A9 (S100 calcium-binding protein A9), VCAM (vascular cell adhesion molecule), CD163 (cluster of differentiation 163), SERPINA1 (serpin family A member 1), ANXA1 (annexin A1), PGAM1 (phosphoglycerate mutase 1), C4A (complement component 4), C1QC (complement C1q C chain), IL-8 (interleukin 8), IL-37 (interleukin 37), AXIN 1 (axis inhibition protein 1), SIRT2 (NAD-dependent deacetylase sirtuin 2), IL-4 (interleukin 4), IL-6 (interleukin 6), IL-10 (interleukin 10), IL-13 (interleukin 13), TNF-α (tumor necrosis factor alpha), IGF-1 (insulin-like growth factor 1), SP (substance P), NPY (neuropeptide Y), MMP-3 (matrix metalloproteinase-3), CX3CL1 (chemokine (C-X3-C motif) ligand 1), and CSF (cerebrospinal fluid).

Peripheral/Central Inflammation	Biomarker	Gene/ Protein	Localization	References
	CCL2	Protein	Plasma	[9]
	*S100A8*, *S100A9*, *VCAM*, *CD163*, *SERPINA1*, and *ANXA1*	Gene	Peripheral B cells	[14]
Peripheral	IL-8, IL-37, AXIN1, and SIRT2	Protein	Serum	
	CRP	Protein	Plasma	[123,124,125,126,127]
	Transferrin; α-, β-, and γ-fibrinogen chains; profilin-1; transaldolase; PGAM1; apolipo-protein-C3; complement C4A and C1QC; immunoglobulin components; and acute phase reactants	Protein	Plasma, serum, and saliva	[130]
	IL-4, IL-6, IL-10, IL-13, and TNF-α	Protein	Plasma	[131,132,133,134,135]
Central	IGF-1	Protein	Serum	[136]
	SP, NPY, MMP-3	Protein	CSF	
	IL-8	Protein	CSF	[137]
	IL-8	Protein	Plasma	[138]
	CX3CL1	Protein	CSF	

## Data Availability

No new data were generated.

## Abbreviations

The following abbreviations are used in this manuscript:

5-HT	Serotonin
5-HT2A	Serotonin 5-HT2A receptor
5-HT2C	Serotonin 5-HT2C receptor
5-HT3	Serotonin 5-HT3 receptor
ACR	American College of Rheumatology
ANXA1	Annexin A1
AP-1	Activator protein 1
ASIC	Acid-sensing ion channel
AXIN1	Axis inhibition protein 1
BBB	Blood–brain barrier
BDNF	Brain-derived neurotrophic factor
C1QC	Complement C1q C chain
C4A	Complement component 4
CB1	Cannabinoid receptor 1
CB2	Cannabinoid receptor 2
CCL13	Chemokine (C-C motif) ligand 13
CCL2	Chemokine (C-C motif) ligand 2
CCL4	Chemokine (C-C motif) ligand 4
CD163	Cluster of differentiation 163
CNS	Central nervous system
CoQ_10_	Coenzyme Q_10_
CRP	C-reactive protein
CSF	Cerebrospinal fluid
CSU	Chronic spontaneous urticaria
CX3CL1	Chemokine (C-X3-C motif) ligand 1
D2	Dopamine D2 receptor
DII	Dietary Inflammatory Index
DNA	Deoxyribonucleic acid
DRG	Dorsal root ganglia
ELISA	Enzyme-linked immunosorbent assay
ERK	Extracellular signal-regulated kinase
EULAR	European Alliance of Associations for Rheumatology
FBD	Functional bowel disorder
FMT	Fecal microbiota transplantation
FODMAP	Fermentable oligo-, di-, and mono-saccharides and polyols
GDNF	Glial-cell-line-derived neurotrophic factor
JNK	c-Jun N-terminal kinase
IBS	Irritable bowel syndrome
IGF-1	Insulin-like growth factor 1
IL-10	Interleukin 10
IL-13	Interleukin 13
IL-17	Interleukin 17
IL-1β	Interleukin 1 beta
IL-21	Interleukin 21
IL-22	Interleukin 22
IL-37	Interleukin 37
IL-4	Interleukin 4
IL-6	Interleukin 6
IL-8	Interleukin 8
IGUBAC-Diet^®^	Inflammatory gut–brain axis control diet
MMP-3	Matrix metalloproteinase-3
MOR	μ-opioid receptor
NA	Noradrenaline
NF-κB	Nuclear factor kappa-light-chain-enhancer of activated B cells
NMDA	N-methyl-D-aspartate
NPY	Neuropeptide Y
NRI	Selective noradrenaline reuptake inhibitor
NSAID	Non-steroidal anti-inflammatory drug
p38 MAPK	p38 mitogen-activated protein kinase
PGAM1	Phosphoglycerate mutase 1
RAGE	Receptor for advanced glycation end products
RCT	Randomized controlled trial
RLS	Restless leg syndrome
RNA	Ribonucleic acid
RT-qPCR	Reverse transcription-quantitative polymerase chain reaction
S100	S100 protein
S100A8	S100 calcium-binding protein A8
S100A9	S100 calcium-binding protein A9
SERPINA1	Serpin family A member 1
SIRT2	NAD-dependent deacetylase sirtuin 2
SNRI	Serotonin-noradrenaline reuptake inhibitor
SP	Substance P
SS	Symptom Severity
SSRI	Selective serotonin reuptake inhibitor
TCA	Tricyclic antidepressant
TLR4	Toll-like receptor 4
TNF-α	Tumor necrosis factor alpha
TRPV	Transient receptor potential vanilloid
VCAM	Vascular cell adhesion molecule
VGCC	Voltage-gated calcium channel
WPI	Widespread Pain Index
